# Editorial: Diabetes and heart failure: basic, translational, and clinical research

**DOI:** 10.3389/fphys.2023.1211232

**Published:** 2023-05-10

**Authors:** Laurent Metzinger, Markus Wallner, Claudio de Lucia

**Affiliations:** ^1^ HEMATIM UR 4666, CURS, University of Picardie Jules Verne, Amiens, France; ^2^ Division of Cardiology, Medical University of Graz, Graz, Austria; ^3^ Lewis Katz School of Medicine, Temple University, Cardiovascular Research Center, Philadelphia, PA, United States; ^4^ Geriatric Center Frullone, ASL (Azienda Sanitaria Locale–Local Health Authority) Napoli 1 Centro, Naples, Italy

**Keywords:** diabetes, heart failure, sacubitril/valsartan, HFrEF–heart failure with reduced ejection fraction, HFpEF–heart failure with preserved ejection fraction, SGLT2 (sodium-glucose cotransporter 2) inhibitor, multiomics, translational reseach

Diabetes mellitus (DM) and heart failure (HF) are complex and systemic diseases that often coexist and share pathophysiological pathways. DM and HF are clinically and pathophysiologically interdependent, such that worsening of one condition is frequently accompanied by worsening of the other. Patients with type 2 DM (T2DM) show a higher risk for developing HF while hospitalization as well as prognosis are worse for patients with DM and HF compared to non-diabetic HF patients. Therefore, there is an increasing unmet need to identify new molecular mechanisms involved in the development and progression of DM complications including heart disease and find specific clinical features in diabetic patients with HF.

Sacubitril/Valsartan (Sac/Val), an angiotensin receptor-neprilysin inhibitor, has been previously demonstrated to improve cardiac function and outcomes in patients with HF with reduced HF (HFrFE) and is currently under investigation to identify further applications in patients with heart disease. Armentaro et al. (*Long Term Metabolic Effects of Sacubitril/Valsartan in Non-Diabetic and Diabetic Patients With Heart Failure Reduced Ejection Fraction: A Real Life Study*) showed an improvement in metabolic profile in patients with HFrEF treated with Sacubitril/Valsartan (Sac/Val). Thirty month-long treatment with Sac/Val ameliorated glycometabolic parameters such as HbA1c, fasting glucose/insulin, IGF-1, the HOMA index, and LDL-cholesterol. In line with previously published data, the authors also found that long-term Sac/Val treatment improved renal function, NTpro-BNP levels, and echocardiographic parameters in HF patients. Moreover, Sac/Val significantly reduced the use of oral antidiabetic drugs and insulin in diabetic patients with HF. Li et al. (*Assessment of ultra-early administration of sacubitril valsartan to improve cardiac remodeling in patients with acute myocardial infarction following primary PCI: rational and design of a prospective, multicenter, randomized controlled trial*) discussed the hypothesis, study objective, inclusion criteria, design and outcome definition of an ongoing, prospective, multicenter, randomized controlled clinical trial aimed to assess the effects of an ultra-early administration of Sac/Val on cardiac remodeling in patients with acute myocardial infarction following primary percutaneous coronary intervention.

Heart failure with preserved ejection fraction (HFpEF) has a high prevalence in the population and accounts at least for half of all patients with HF. Dhore-patil et al. (*Diabetes mellitus and heart failure with preserved ejection fraction: role of obesity*) analyzed the characteristics of the metabolic phenotype (DM-obesity) of HFpEF that is the most common in the clinical practice, with poor clinical outcomes and an urgent need for effective treatments. The authors examined how obesity and diabetes induce the development and progression of left ventricular remodeling. Pathophysiological mechanisms involved in the HFpEF phenotype include low-grade systemic inflammation, microvascular dysfunction and increase in visceral as well as pericardial/epicardial adipose tissue. In this review article, recent therapeutic advances were discussed, including glucagon-like peptide-1 receptor agonists (GLP-1 RAs), Sodium-glucose co-transporter 2 inhibitors (SGLT2i) and metabolic surgery. Ali et al. (*Temporal trends in outcomes of ST-elevation myocardial infarction patients with heart failure and diabetes*) investigated the temporal trends in demographics and outcomes in diabetic heart failure patients admitted with STEMI utilizing data from the national inpatient sample (NIS) database between 2005–2017. The mean age of patients with HFrEF and the rate of hospitalization for STEMI in this cohort decreased over time although the prevalence of traditional risk factors increased. In contrast, in patients with HFpEF hospitalization rates for STEMI steadily increased. Mortality rates remained stable in both HF entities with lower rates in patients with HFpEF compared to HFrEF. These findings in temporal trends are consistent with other studies and underpin that HF is a major public health problem with a significant financial and societal burden.

Mekhaimar et al. (*Diabetes outcomes in heart failure patients with hypertrophic cardiomyopathy*) investigated the prevalence and outcomes of diabetes in patients with hypertrophic cardiomyopathy. Data derived from the national inpatient sample (NIS) database between 2005–2015 a large all-payer database in the US. Almost one-third of patients with HCM had diabetes with an increasing prevalence over time, which corresponds to a general increase of diabetes and other traditional cardiovascular risk factors in the general population. Diabetes was associated with a lower risk of in-hospital mortality but an increased length of stay and total charges/stay. There are conflicting data with regards to a lower in-hospital mortality with diabetes. This finding cannot be easily explained. Further trial evidence is warranted to find conclusive answers to this question. Kreiner et al. (*The potential of glucagon-like peptide-1 receptor agonists in heart failure*) analyzed the beneficial effects of GLP-1 RAs in patients with DM and HF. In the last two decades, GLP-1 RAs have been shown to improve glycemic control in T2DM and to decrease body weight in subjects with overweight/obesity. Interestingly, GLP-1 RAs have also been recommended for patients with T2DM and cardiovascular diseases (CVDs) to improve CV outcomes. Moreover, this review article discussed the use of GLP-1 RA treatment for people with T2DM and HF particularly when SGLT2i are not well tolerated. Finally, the authors suggested that the cardioprotective effect of GLP-1 RAs may be particularly pronounced in patients with HFpEF and metabolic comorbidities such as obesity. Currently ongoing clinical trials are testing this hypothesis*.*

Tayanloo-Beik et al. (*Diabetes and heart failure: multi-omics approaches)* reviewed recent approaches used to detect specific molecular and cellular pathways involved in both HF and T2DM using a multiomics approach. They concentrated on oxidative stress with a particular focus on diminished myocardial perfusion linked to endothelial dysfunction, dysregulated glucose levels due to insulin resistance and reactive oxygen species (ROS) mediation. The main molecular targets discussed were protein phosphatase, sarcoplasmic/endoplasmic reticulum Ca2+ ATPase 2a and phosphorylated SERCA2a. This paper is indicative of the impact that the new omics technologies will bring to the table in the comprehension of the molecular pathways implicated in HF and T2DM.

Ge et al. (*The Serum Soluble Scavenger with 5 Domains Levels: A Novel Biomarker for Individuals with Heart Failure*) studied correlations between seric levels of the Soluble Scavenger with 5 Domains (SSC5D) and HF in a cohort of 276 HF patients or normal controls. Additionally, Ssc5d mRNA levels were quantified in murine heart tissue after cardiovascular disorder occurred. They found that SSC5D levels were significantly increased in pathological murine hearts compared to control. Accordingly, seric SSC5D levels were elevated in the HF group compared with healthy patients. Finally, they found that serum SSC5D levels were positively correlated with N-terminal pro-B-type natriuretic peptide while they were inversely correlated with left ventricular ejection fraction. Thus, this interesting study provides evidence indicating that SSC5D seric levels may be a novel biomarker for patients with HF.

Hebbard et al. (*Diabetes, heart failure, and COVID-19: an update*) endeavored to review the interactions between the severe acute respiratory syndrome coronavirus 2 (SARS-CoV-2) pandemic, HF and T2DM. It is widely known that, when infected with SARS-CoV-2, there is a more severe prognoses for patients with existing cardiovascular disease than healthy counterparts. What is less known is that newly published studies hint at a correlation between SARS-CoV-2 infection and an enhanced incidence of new-onset HF and T2DM, regardless of disease severity. The authors interestingly point out new potential mechanisms that may explain how SARS-CoV-2 infection would trigger HF and T2DM in heretofore unaffected patients such as systemic inflammation, cytokine storm, hyperglycemia *etc.* This interesting review points out clues to use innovative ways to optimize old treatments to develop new preventative measures for patient care.

We hope that the manuscripts published within our Research Topic will contribute to a more accurate risk stratification for diabetic patients with HF and to identify appropriate and timely treatments for subjects with DM and heart disease.

